# Eliminating *Plasmodium falciparum* malaria: results from tailoring active case detection approaches to remote populations in forested border areas in north-eastern Cambodia

**DOI:** 10.1186/s12936-021-03622-4

**Published:** 2021-02-22

**Authors:** Ann-Sophie Stratil, Lieven Vernaeve, Sergio Lopes, Yves Bourny, Kylie Mannion, Prudence Hamade, Arantxa Roca-Feltrer, James K. Tibenderana, Siv Sovannaroth, Mark Debackere

**Affiliations:** 1 Malaria Consortium, London, UK; 2 Malaria Consortium, Phnom Penh, Cambodia; 3 National Center for Parasitology, Entomology and Malaria Control (CNM), Phnom Penh, Cambodia

**Keywords:** Malaria elimination, Forest malaria, Early diagnosis and treatment, Case detection, Community health worker, *Plasmodium falciparum*, Mobile and migrant populations, Mobile malaria worker, Greater mekong subregion, Regional artemisinin initiative

## Abstract

**Background:**

Remaining *Plasmodium falciparum* cases in Cambodia are concentrated in forested border areas and in remote populations who are hard to reach through passive case detection. A key approach to reach these populations is active case detection by mobile malaria workers (MMWs). However, this is operationally challenging because of changing movement patterns of the target population moving into less accessible areas. From January 2018 to December 2020, a tailored package of active case detection approaches was implemented in forested border areas of three provinces in north-eastern Cambodia to reach remote populations and support the elimination of falciparum malaria.

**Methods:**

Key elements of this project were to tailor approaches to local populations, use responsive monitoring systems, maintain operational flexibility, build strong relationships with local communities, and implement close supervision practices. MMWs were recruited from local communities. Proactive case detection approaches included mobile malaria posts positioned at frequented locations around and within forests, and locally informed outreach activities targeting more remote locations. Reactive case detection was conducted among co-travellers of confirmed cases. Testing for malaria was conducted independent of fever symptoms. Routine monitoring of programmatic data informed tactical adaptations, while supervision exercises ensured service quality.

**Results:**

Despite operational challenges, service delivery sites were able to maintain consistently high testing rates throughout the implementation period, with each of 45 sites testing a monthly average of 64 (SD 6) people in 2020. In 2020, project MMWs detected only 32 *P. falciparum* cases. Over the project period, the *P. falciparum*/*P. vivax* ratio steadily inversed. Including data from neighbouring health centres and village malaria workers, 45% (80,988/180,732) of all people tested and 39% (1280/3243) of *P. falciparum* cases detected in the area can be attributed to project MMWs. Remaining challenges of the last elimination phase include maintaining intensified elimination efforts, addressing the issue of detecting low parasitaemia cases and shifting focus to *P. vivax* malaria.

**Conclusions:**

Reaching remote populations through active case detection should remain a key strategy to eliminate *P. falciparum* malaria. This case study presented a successful approach combining tailored proactive and reactive strategies that could be transferred to similar settings in other areas of the Greater Mekong Subregion.

## Background

Cambodia has made huge progress towards eliminating *Plasmodium falciparum* malaria in recent years and is well on track to achieve its goal by the end of 2023 [1, 2]. This can be attributed to intensified efforts to accelerate the elimination of falciparum malaria in the Greater Mekong Subregion (GMS) under the two consecutive Regional Artemisinin-resistance Initiative (RAI) programmes since 2016.

In Cambodia, the RAI programme focuses on international borders and forest areas where remaining malaria parasite reservoirs are clustered [3–5]. In these areas, the population at highest risk of malaria infection are mobile and migrant populations and forest workers, who often spend several nights in the forest at a time or even stay there permanently [5, 6]. These populations are hard to reach via the common community-based passive case detection system. This system requires people to actively seek care with village malaria workers (VMWs) while mobile and migrant populations might not know about the local health system or not be knowledgeable about malaria symptoms and appropriate health-seeking behaviour if they migrated from other regions or non-malaria endemic areas [7–9]. Expanding access to early diagnosis and effective treatment (EDAT) is a key element of the Cambodia National Strategic Plan for Elimination of Malaria [10]. In 2009, the National Center for Parasitology, Entomology and Malaria Control (CNM) introduced the community health worker role of mobile malaria workers (MMWs) to target these remote populations with active case detection approaches [8]. In border areas, this is challenging due to highly heterogeneous micro-geographical epidemiology, unexplored forest areas, difficult to access terrain and changing movement and behavioural patterns of populations.

One component under the under the second RAI programme (RAI2E) 2018–2020 was a project delivering a tailored package of active case detection approaches through MMWs in selected hard-to-reach border areas in north-eastern Cambodia. The approach was developed in alignment with the Cambodia National Strategic Plan for Elimination of Malaria [10], in close collaboration with the CNM. The RAI2E grant concluded by the end of 2020 and was followed by RAI3, the third 3-year-grant-cycle in the GMS. The conclusion of the second grant cycle was an appropriate occasion to look back on the achievements during the RAI2E implementation period, to investigate challenges faced along the way, and to discuss priorities for the final phase of malaria elimination.

## Objectives

This case study aimed (1) to outline the approach to deliver a tailored package of active case detection approaches with the aim of providing EDAT services to remote populations in border areas of three provinces in north-eastern Cambodia under the RAI2E programme, and (2) to describe results achieved and discuss remaining challenges for the last malaria elimination phase.

## Methods

### Project setting

The project started in January 2018 and ended in December 2020. It was implemented in border areas of the three Cambodian provinces Preah Vihear, Stung Treng and Ratanakiri along the Laos, Thailand and Vietnam borders. Border areas in these provinces are forested and difficult to access, especially during the rainy season between May and October. The existing network of eight to ten MMWs working in the area prior to 2018 was scaled up to 50 MMWs as part of the project. MMWs work under the CNM and are assigned to specific health centres that oversee the activities of their assigned community health workers (MMWs and VMWs) and provide them with treatment supplies. The target population was everyone living and working within the catchment area of the project sites with a focus on reaching the high risk population of adult males over the age of 15 as defined by the Malaria Elimination Action Framework 2016–2020 [11].

### Project design

The goal of this project was to contribute to the RAI2E objective of accelerating falciparum malaria elimination. The overall approach was to implement a tailored package of active case detection approaches through a network of MMWs in order to reach remote populations and deliver EDAT services in line with national treatment guidelines [12]. The logic model of the project assumed that the efficient delivery of the package of tailored active case detection approaches in combination with quality case management would lead to effective detection and treatment of *P. falciparum* cases among target populations, contributing to the accelerated elimination of falciparum malaria in project areas.

The technical design of the project built on learnings from a project implemented under the first RAI programme 2016–2017. Said project had initially started positioning MMWs at screening points at official and unofficial border crossings [13]. Based on insights on population movements and refusal rates, this approach shifted towards positioning MMWs at mobile malaria posts and conducting outreach activities to reach mobile and migrant populations and forest workers. The lessons from this operational experience and other research projects in the same region can be summarized into five guiding principles that informed the design of the RAI2E project: (i) the need to tailor case detection approaches to the local target population and context, (ii) using responsive monitoring systems, (iii) maintaining operational flexibility, (iv) building strong community relationships, and (v) closely supervising the MMW network.

### Translation into practice

All project activities were aligned with the National Strategic Plan for Elimination of Malaria 2011–2025 and the Malaria Elimination Action Framework 2016–2020 and were developed and delivered in close collaboration with the CNM [10, 11]. As per CNM guidelines, MMWs were directly recruited from local target communities (i.e. forest-goers or mobile and migrant populations) in high risk (i.e. annual parasite index > 5 per 1000 population based on data from the national malaria information system, MIS) and remote areas (i.e. more than 5 kms or more than one hour walk from the nearest health centre) where no other VMWs or MMWs worked. MMWs were trained on service delivery and case management in collaboration with the CNM which also conducted regular supervision visits. They were paid USD 20 per month by the CNM according to national guidelines, and an additional USD 5 per day for each workday through the RAI2E project.

The package of active case detection approaches consisted of proactive case detection delivered through mobile malaria posts and outreach activities, and occasional reactive case detection among co-travellers of index cases. MMWs were assigned to work either at a mobile malaria post or an outreach site from where to conduct outreach activities to different locations. Where needed, two MMWs were assigned to work at the same service delivery site with alternating shifts. Mobile malaria posts were placed at border crossings, at forest entry points or market places close to locations where the national MIS had reported high caseloads of *P. falciparum* or mixed (*P. falciparum* + second species) cases and where target populations frequently passed by on their way to and from the forest. MMWs positioned at mobile malaria posts approached everyone that passed their post for testing. Locations for outreach activities were selected by MMWs based on local knowledge about the area and usually targeted areas close to or within the forest not covered by mobile malaria posts or where communities could not easily access health services (such as remote forests and workplaces). During an outreach activity, MMWs would travel to the selected destination from their outreach site, and offer testing to everyone present at the location. Additionally, where feasible, MMWs were asked to conduct reactive case detection if a confirmed malaria case had spent time in the forest in the previous two weeks. This consisted of identifying and testing co-travellers of the confirmed index case for malaria. Active door-to-door screening was conducted in Preah Vihear in 2020 to confirm no new *P. falciparum* cases.

Operational targets for mobile malaria posts were to test 20 people per week and for outreach sites to conduct two outreach activities per week. Targets were set based on experiences from the first RAI programme and feasibility considerations based on the available budget. In general, mobile malaria posts operated seven days per week with standard operating hours between 7 am and 7 pm with operational flexibility to adjust to local population movement patterns. These EDAT services were complemented by distributing long-lasting insecticidal nets and hammock nets (LLINs/LLIHNs) as well as conducting health education among target communities as prescribed by the CNM.

Tests and treatment supplies were provided by associated health centres through the CNM. Malaria tests were conducted with *P. falciparum/Plasmodium vivax* rapid diagnostic tests (RDTs) (SD BIOLINE Malaria Ag P.f/P.v, Standard Diagnostics) applied as per product guidelines [18] without prior screening for fever. Uncomplicated *P. falciparum*, mixed and *P. vivax* cases were treated with artemisinin-based combination therapy (ACT), where feasible and referred to associated health centres in case of treatment stock-outs, severe cases or contraindications in line with national guidelines [12]. First-line ACT was artesunate-mefloquine with pyronaridine-artesunate used during prolonged stock-out of first line treatment. Single low-dose primaquine was given to *P. falciparum* and mixed cases.

The project further employed nine community mobilization officers placed at field sites to directly support MMWs, a field implementation manager placed at a coordination office and a technical specialist. The team was responsible for ensuring the quality and efficiency of project delivery through monthly assessments and supervision of service delivery by MMWs with standardized checklist, and responsive monitoring. The aim of responsive monitoring was to sustain and improve service delivery efficiency and effectiveness in terms of achieving high testing rates and detecting *P. falciparum* cases, and ensuring constant availability of test and treatment supplies. To achieve this, (i) quantitative evidence including testing rates, case clusterings, and stock information, (ii) operational experience about the feasibility of operating service delivery sites in specific areas, and (iii) local knowledge about frequented forest areas and population movements were regularly reviewed by the project team to recognize emerging needs early on. These insights were used to continuously adapt the positioning of mobile malaria posts, target locations of outreach activities and other operational aspects in collaboration with the CNM, local authorities and other implementing partners.

#### Tailoring service delivery to the local target population and context

Efficient active case detection approaches have to consider the whole spectrum of the target population. Remote populations consist of various subpopulations, including local and mobile forest workers, construction workers, security personnel and border-crossers, who are each involved in different forest-related activities [9]. Active case detection approaches targeting these populations have to be tailored to each subpopulation individually leveraging local knowledge on population behaviour, movements and work sites. Reactive approaches have to target individuals sharing the same exposure time and location as the index case, i.e. co-travellers rather than household members [13–15].

#### Using responsive monitoring systems

Responsive monitoring systems are a key component to efficiently target interventions. Hotspots and population movements change constantly which requires routinely adjusting and optimizing operational timing and locations of testing [16, 17]. This can only be achieved by systematic monitoring practices facilitating collaborative decisions between all relevant stakeholders based on quantitative evidence, operational experience and local insights.

#### Maintaining operational flexibility

In order to respond to the findings from the responsive monitoring system in a timely manner, the operational set-up must allow the flexible adaptation of project activities.

#### Building strong community relationships

Any approach to reach remote populations must involve strong and sustained relationships with these communities. A key factor in achieving this is the recruitment of MMWs directly from the target communities of forest-goers and mobile and migrant populations which is essential to establish them as trusted service providers based on existing relationships and expert knowledge about the local environment [10].

#### Facilitating close supervision systems

Closely supervising the MMW network is needed to ensure quality of service delivery and to sustain motivation among health workers. This includes routine supervision practices by national and project staff, as well as regular meetings for MMWs to share their experiences and knowledge. Operationally, this means it is necessary to factor extensive travel by project staff in difficult road conditions and proper deployment of staff to guarantee effective operations, particularly during the rainy season.

### Data collection and analysis

This case study focused on the implementation period between 2018 and 2020 including data from 1st January 2018 until 21st of December 2020 which corresponded to the reporting period of the national MIS. The first COVID-19 case was diagnosed in Cambodia on 27th January 2020. As high testing rates were maintained throughout 2020, results were reported under the assumption that the health-seeking behaviour of the target communities had not changed. Data analysis was based on routine programme data recorded by MMWs. Data was entered into Excel and Microsoft Access databases by community mobilization officers monthly, and integrated and verified by the field implementation manager. MIS data from health centres associated with project MMWs and data from VMWs associated with the same centres was provided by the CNM. Quantitative data were summarized descriptively using Microsoft Power BI Desktop (version 2.85.682.0) and Microsoft Excel 2016 (version 16.0.5095.1000). Information on operational considerations and challenges were summarized based on project reports compiled by project staff. The term *P. falciparum* cases includes *P. falciparum* and mixed cases.

Results were summarized to outline the operational aspects of the project including the set-up, efficiency and quality of service delivery, the effectiveness of active case detection approaches in reaching the target population and detecting new *P. falciparum* cases and the contribution of the project to the accelerated elimination of falciparum malaria in project areas.

## Results and implementation

### Operational set-up

**Fig. 1:**
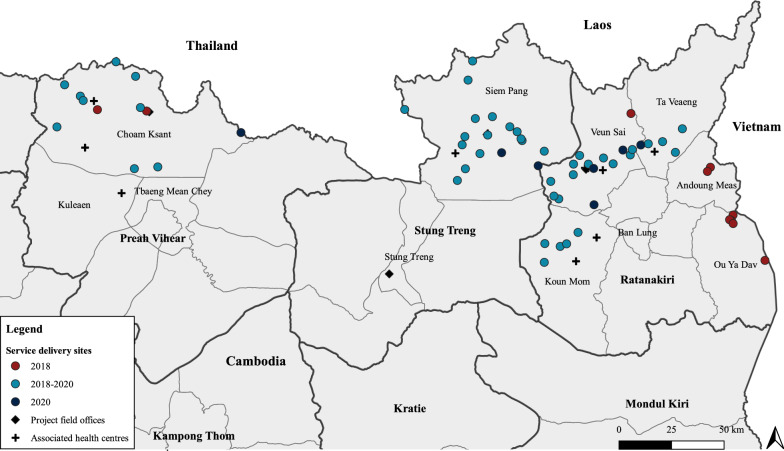
Service delivery sites between 2018 and 2020

At the start of the project in January 2018, the goal agreed among stakeholders was to recruit 32 MMWs and deploy them at service delivery sites in border areas of the provinces Preah Vihear, Ratanakiri and Stung Treng. Due to delays in the official project start, services were delivered through 23 service delivery sites until June 2018. After the official project start, the number of service delivery sites was quickly scaled up to the intended 32 sites and further expanded to 45 service delivery sites operated by 50 MMWs by November 2018. This expansion of services was coordinated with local authorities and other implementing partners to avoid overlapping of service delivery. Initial location of service delivery sites was based on risk areas identified during the first RAI programme. Over the project period, responsive monitoring facilitated targeted relocations to increase the efficiency of service delivery (Fig. 1).

### Efficiency of service delivery

**Fig. 2:**
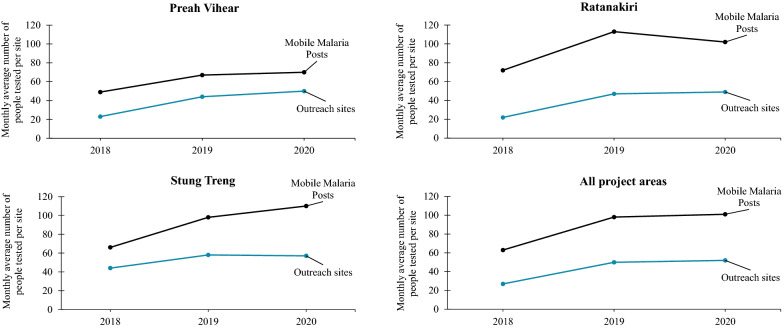
Monthly average of people tested per site by service delivery type between 2018 and 2020

Efficiency of service delivery was quantified through the monthly average number of people tested per site (Table 1). While in the first project year 2018 a monthly average of only 37 (standard deviation, SD 13) people was tested per site, improving the placement of mobile malaria posts and outreach activities as well as operational timings increased the monthly average number of people tested per site in the subsequent project years to 62 (SD 14) in 2019 and 64 (SD 6) in 2020. Across project years, mobile malaria posts tested a monthly average of approximately twice as many people per site as outreach sites due to their continuous operations throughout the month while outreaches were point-in-time activities only taking place twice a week (Fig. 2). The monthly average of people tested per service delivery site differed between provinces with a lower monthly average in Preah Vihear due to a decline in local forest-goer activities in this province as a result of increased forest protection. The overall daily average of people tested taking into account only operational days was 4 (SD 1) people for both mobile malaria posts and outreach sites, showing that, overall, mobile malaria posts and outreach activities were equally efficient in testing people per operational day.

**Table 1:** Monthly average of service delivery sites and monthly average number of people tested between 2018 and 2020

Province	Project year	Mobile malaria posts		Outreach sites		All types of service delivery sites	
		Monthly average number of service delivery sites (SD)	Monthly average number of people tested (SD)	Monthly average number of service delivery sites (SD)	Monthly average number of people tested (SD)	Monthly average number of service delivery sites (SD)	Monthly average number of people tested (SD)
Preah Vihear	2018	2 (0)	49 (33)	6 (1)	23 (8)	8 (1)	30 (14)
	2019	2 (0)	67 (20)	7 (0)	44 (14)	9 (0)	49 (14)
	2020	2 (0)	70 (31)	4 (1)	50 (9)	6 (1)	57 (13)
Ratanakiri	2018	2 (1)	72 (39)	10 (4)	22 (13)	12 (5)	31 (15)
	2019	4 (0)	113 (33)	16 (0)	47 (10)	20 (0)	60 (14)
	2020	4 (0)	102 (13)	19 (1)	49 (7)	23 (1)	58 (7)
Stung Treng	2018	3 (1)	66 (18)	5 (4)	44 (16)	8 (5)	59 (14)
	2019	5 (0)	98 (28)	11 (0)	58 (11)	16 (0)	70 (16)
	2020	5 (0)	110 (15)	12 (0)	57 (7)	17 (0)	73 (8)
All project areas	2018	8 (2)	63 (24)	20 (7)	27 (13)	28 (9)	37 (13)
	2019	11 (0)	98 (26)	34 (0)	50 (11)	45 (0)	62 (14)
	2020	11 (1)	101 (12)	35 (1)	52 (6)	45 (1)	64 (6)

Strategies to increase the monthly average of people tested included tactically relocating service delivery sites in close collaboration with local authorities and allowing MMWs to flexibly adapt their daily operations to align with forest-goer movements. In 2020, mobile malaria posts with lower testing rates operated as a mobile malaria post in the morning and conducted outreach activities in the evening, thereby improving the reach of their services. One strategy to respond to population movement changes in a timely and responsive manner was the development of a ‘seasonality calendar’: through a workshop held in December 2018, project teams worked with MMWs to develop province-specific calendars detailing when mobile and migrant populations usually participated in activities that increased the risk of malaria infection, including the shifts of construction and work sites within forests based on seasonality. The calendar was used by project staff to ensure that service delivery sites were positioned at the right place at the right time.

### Quality of case management

Table 2 depicts the number of people tested, cases detected and proportion of detected cases treated and referred by project year. The tailored package of active case detection approaches facilitated the testing of 80,988 people for malaria and the detection of 4385 malaria cases between January 2018 and December 2020.

**Table 2:** Details on number of tests, cases detected and proportion of cases treated or referred between 2018 and 2020

Project year	People tested *n* (column %)	Malaria cases detected *n* (row %)		Proportion of malaria cases that received treatment or were referred n (%)		
		*P. falciparum*	*P. vivax*	ACT^a^	Referred^b^	Primaquine^c^
2018	13,407 (17%)	934 (42%)	1264 (58%)	1739 (79%)	459 (21%)	81 (9%)
2019	33,300 (41%)	314 (19%)	1309 (81%)	1499 (92%)	124 (8%)	159 (51%)
2020	34,281 (42%)	32 (6%)	532 (94%)	517 (92%)	47 (8%)	26 (81%)
Overall	80,988 (100%)	1280 (30%)	3105 (71%)	3755 (86%)	630 (14%)	266 (21%)

^a^Proportion of all detected malaria cases who were treated with artemisinin-combination therapy (ACT)

^b^Proportion of all detected cases who were referred for any reason

^c^Proportion of *P. falciparum* cases who were treated with single low-dose primaquine

Of detected malaria cases (all *Plasmodium* species), 86% (3755/4385) were treated with ACT as per national guideline, while the remaining 14% (630/4385) were referred to the nearest health centre to receive appropriate treatment. In the second and third quarter of 2018, primary service delivery sites experienced stock-outs of first and second line treatment supplies: only 79% (1739/2198) of detected malaria cases (all *Plasmodium* species) received ACT treatment at site while 21% (459/2198) of cases were referred to nearby health centres in 2018. Referral costs could be covered by project savings, but success of referral was not followed up, so actual reception of treatment could not be confirmed. During this period, a project implemented by Médecins Sans Frontières in the neighbouring district Chey Saen provided treatment supplies to support the sustainment of service delivery for a few weeks. Once stock-outs were resolved, the proportion of malaria cases treated directly as per national guideline improved from 79% (1739/2198) in 2018 to 92% (517/564) in 2020.

In the beginning of the project, the appropriate dosage of single low-dose primaquine for *P. falciparum* cases weighing < 50 kg (7.5 mg) was not available in project areas. However, monitoring data showed that a large proportion of *P. falciparum* cases in the project area actually required this dosage: 27% (41/154) of adult *P. falciparum* cases (≥ 15 years old) with available weight data and 43% (85/200) of all *P. falciparum* cases with available weight data detected in 2018 weighed < 50 kg. After this issue was raised to the CNM and the appropriate dosage was supplied to project areas in the second half of 2019, the proportion of *P. falciparum* cases treated with primaquine as per national guidelines improved from 9% (81/934) in 2018 to 81% (26/32) in 2020.

### Reaching the target population

**Fig. 3:**
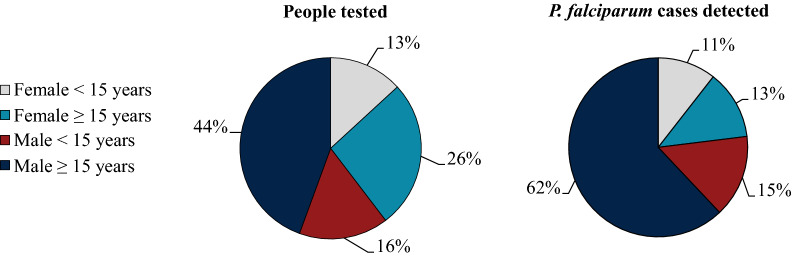
Age and gender composition of people tested and detected *P. falciparum* cases between 2018 and 2020

Figure 3 shows the gender and age composition of the population that was tested and *P. falciparum* cases detected. People tested through the tailored active case detection package were predominantly male (60%, 48,894/80,988) and at least 15 years old (71%, 57,396/80,988). Specifically, males who were at least 15 years old made up 44% (35,967/80,988) of the total population tested and 62% (794/1280) of *P. falciparum* cases, while females who were at least 15 years old made up 26% (21,429/80,988) of all people tested and 13% (160/1280) of *P. falciparum* cases. 26% (326/1280) of *P. falciparum* cases were children under 15 years old.

### Effectiveness of case detection approaches in detecting *P. falciparum* cases

Table 3 describes the number of people tested to find 1 *P. falciparum* case by case detection approach. The number of people tested to find 1 *P. falciparum* case is the ratio of the number of people tested to the number of *P. falciparum* cases detected in a specified period. It is an indicator for the ‘effectiveness of a case detection approach’ compared to other approaches in similar settings and periods.

**Table 3:** Number of people tested to find 1 P. falciparum case from January 2018 and December 2020

Project year	Case detection approach	*P. falciparum* cases detected	Number of people tested	Number of people tested to find 1 *P. falciparum* case^a^
2018	Mobile malaria post	545	5897	11
	Outreach	343	6906	20
	Co-traveller investigation	46	604	13
2019	Mobile malaria post	136	12,839	94
	Outreach	170	19,693	116
	Co-traveller investigation	8	768	96
2020	Mobile malaria post	12	12,421	1035
	Outreach	20	21,389	1069
	Co-traveller investigation	–	123	–
	Active screening	–	348	–
Overall	Mobile malaria post	693	31,157	45
	Outreach	533	47,988	90
	Co-traveller investigation	54	1495	28
	Active screening	–	348	–

*P. falciparum* cases detected during the specific period

^a^Number of all tests conducted in a specified period divided by number of

Overall, mobile malaria posts tested an average of 45 people to find 1 *P. falciparum* case (31,157 people tested and 693 *P. falciparum* cases detected), while an average of 90 people were tested to find 1 *P. falciparum* case through outreach activities (47,988 people tested and 533 *P. falciparum* cases detected). The number of people tested to find 1 case increased notably by project year which was driven by the decrease in total number of *P. falciparum* cases in the project area from 2018 to 2020. In each individual project year, mobile malaria posts needed to test less people to find 1 *P. falciparum* case than outreach activities, indicating that mobile malaria posts were more effective in detecting new *P. falciparum* cases than outreach activities in the given local context.

Between 2018 and 2020, an average of 28 co-travellers were tested to find 1 *P. falciparum* case (1495 people tested and 54 *P. falciparum* cases detected) and no *P. falciparum* cases were found through active door-to-door screening (348 people tested). However, it should be taken into account that these two case detection approaches were conducted at a much smaller scale than mobile malaria posts and outreaches, hence limiting the comparability of the numbers of people tested to find 1 *P. falciparum* case.

### Contribution to accelerated elimination of falciparum malaria in project areas

**Fig. 4:**
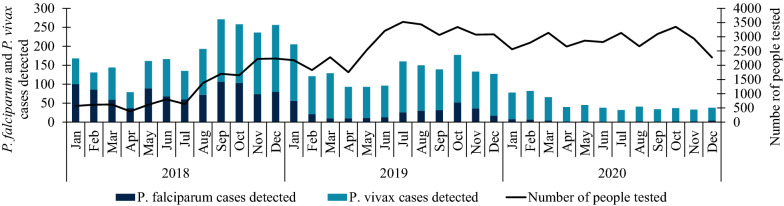
Number of *P. falciparum* cases and *P. vivax* cases detected and number of people tested in project areas between 2018 and 2020

Figure 4 shows the absolute number of malaria cases detected by species and the number of people tested through project service delivery sites. The number of *P. falciparum* cases detected decreased by 97% from January to December 2018 to the same period in 2020 with an increase in number of people tested per month. Between January and December 2018, 7.4 *P. falciparum* cases were detected for every *P. vivax* case. This ratio had consistently inversed since June 2018, and between January and December 2020, only 0.6 *P. falciparum* cases were detected for every *P. vivax* case which is a significant achievement on the path to falciparum malaria elimination. Between January and December 2020, only 32 *P. falciparum* cases were detected in project areas of Stung Treng and Ratanakiri, while no *P. falciparum* cases had been detected in project areas of Preah Vihear since October 2019.

Taking into account data reported by associated health centres and VMWs in the project area, MMWs under this project have contributed to testing 45% (80,988/180,732) of all people tested and detecting 39% (1280/3243) of all *P. falciparum* cases and 72% (1280/1768) of community *P. falciparum* cases (VMW and MMWs) between 2018 and 2020.

## Discussion

This case study described the results from implementing a tailored package of active case detection approaches to reach remote populations in border areas in three provinces in north-eastern Cambodia between January 2018 and December 2020.

Results showed that service delivery sites efficiently reached and tested people with each of 45 service delivery site testing a monthly average of 64 (SD 6) people in 2020. Cases were managed with high quality, with all detected cases either treated or referred as per national guidelines. Operational challenges (stock-outs of treatment, changing population movement patterns, hard-to-reach operational area) did not lastingly impact the overall quality of service delivery. On the contrary, efficiency of service delivery was improved over the project period through tactical optimization of timing and location of service delivery and possibly an increase of trust between MMWs and local communities. Responsive monitoring and evaluation systems in combination with strong community relationships can contribute to tangible improvements of project delivery and efficiency of services.

Results showed that tailored active case detection approaches indeed resulted in reaching the target population of adult males over the age of 15 as defined by the Malaria Elimination Action Framework 2016–2020 [11] and detecting *P. falciparum* cases. While the group most at risk of being infected showed still to be males over 15 years, people travelled to forests in mixed-age and -gender groups. Hence, programmes need to maintain sufficient stock of paediatric formulations for lower weight individuals and effective referrals of pregnant women to health centres.

Comparing proactive case detection approaches, while mobile malaria posts and outreach activities were equally efficient in reaching people with similar daily averages considering operationally active days, mobile malaria posts needed to test less people on average to find 1 *P. falciparum* case indicating a more effective case detection strategy. The importance of well-functioning mobile malaria posts in any malaria elimination strategy has been previously reported [19]. Nevertheless, outreach activities remain invaluable in order to reach less accessible places and achieve testing targets set by the CNM. Further, outreach activities are beneficial in providing health education and establishing the presence of MMWs in remote communities as they regularly interact with the target population, pass on malaria messages and distribute LLIN/LLIHNs. Through these interactions, members of target communities are encouraged to actively seek out health services from MMWs or other service providers when they develop symptoms. Previous studies have pointed out the importance of community trust in service providers for health-seeking behaviour [20, 21].

As the size of target population had not been formally quantified, this case study could not determine changes in malaria incidence among the target population over time. Despite this limitation, the results presented in this case study showed that MMWs under this project significantly contributed to maintaining high testing rates and detecting new *P. falciparum* cases in the area. Using a tailored package of approaches was shown to be an effective way to find remaining *P. falciparum* cases and contribute to the acceleration of falciparum malaria elimination.

## Remaining challenges

### Maintaining intensified efforts to find last *P. falciparum* cases and prevent re-establishment

Using tailored packages of active case detection approaches in combination with the community-based passive case detection system will remain a key approach for the final phases of the elimination of falciparum malaria. Detecting and treating the last remaining *P. falciparum* cases will continue to require intensive efforts and resources: the results from this case study showed that the number of people tested to find 1 *P. falciparum* case steadily increased over three years of implementation, requiring health workers to remain motivated among decreasing positive case yields. While testing fatigue among the health work force and target population was not observed, targeted health promotion and social and behaviour change communication activities need to be implemented to maintain awareness. At the end of 2020, the CNM started to implement case investigations for every *P. falciparum* case within 3 days of notification to determine if the infection was acquired locally. If it was, a focus investigation was conducted within 14 days of detection to determine the set of interventions to be undertaken [22]. These intensified active case detection and surveillance activities will need to be maintained until malaria is eliminated. Once elimination is achieved, preventing re-establishment will require sustainable approaches to retaining a health worker force with appropriate skill sets to respond to imported or introduced malaria cases. This might warrant the long-term integration of malaria services into larger health system structures [23].

### Addressing the asymptomatic reservoir and the low sensitivity of RDTs

Previous studies in the region have shown high proportions of asymptomatic *P. falciparum* carriers among the risk population [16, 24]. This population is usually missed by conventional RDTs with data showing that sensitivity of RDTs compared to polymerase chain reaction (PCR) in asymptomatic carriers could be as low as 18% [17]. Sensitivity of RDTs among symptomatic cases has been reported to be insufficient as well, especially in cases with low parasitaemia [17, 25, 26]. Hence, many *P. falciparum* carriers, symptomatic and asymptomatic, are missed by field diagnostics while they might still constitute an ongoing reservoir of further transmission. Diagnostic tools with higher sensitivity than conventional RDTs have been trialled, but either were not adopted as in the case mobile PCR laboratories that could have been used in field settings [27] or were shown not to sufficiently increase sensitivity as in the case of high-sensitivity RDTs [22]. Recently, researchers and policymakers in Cambodia discussed novel tools to further accelerate falciparum malaria elimination and considered approaches not depending on a confirmed diagnosis. As mass drug administrations still face substantial operational challenges including the achievement of high coverage and appropriate drug choice, trialling malaria prophylaxis in forest workers was deemed a promising idea to be explored in the short term [22, 28].

### Shifting focus to vivax malaria

As falciparum malaria has been steadily declining, vivax malaria has become more prominent. While the epidemiology of the two malaria parasite species is similar in terms of geography, transmission pattern and risk groups [29], treatment of *P. vivax* malaria poses a particular challenge to elimination efforts due to its potential to cause periodic relapses. National guidelines recommend primaquine radical cure which requires prior testing for glucose-6-phosphate dehydrogenase (G6PD)-deficiency to ensure safe drug administration [12]. While point-of-care tests for G6PD deficiency have been trialled [30], the lack of adoption and availability of these tests prevents the routine use of primaquine radical cure at primary health facilities [29]. Approaches to safely deliver appropriate treatment to *P. vivax* cases need to be developed alongside the continued delivery of tailored *P. falciparum* interventions.

## Conclusions

This case study presented a successful approach to implementing tailored active case detection strategies in forested border areas in north-eastern Cambodia. Key components of the project success were a combination of proactive and reactive case detection activities tailored to the local target population and context, operational flexibility, strong relationships with local communities, close supervision and quality assurance of service delivery and responsive systems that were able to adapt to changing circumstances. While some challenges remain that need to be addressed before malaria elimination can be reached, this is an effective approach which could be transferred to similar geographies and risk populations in other areas of the GMS targeting malaria elimination.

## Data Availability

The data that support the findings of this study are available from the corresponding author upon reasonable request.

## Abbreviations

MMW: Mobile malaria worker
GMS: Greater Mekong Subregion
RAI: Regional artemisinin-resistance initiative
RAI2E: Regional artemisinin-resistance initiative 2 elimination
EDAT: Early diagnosis and effective treatment
CNM: National Center for Parasitology, Entomology and Malaria Control
VMW: Village malaria worker
ACT: Artemisinin-based combination therapy
LLINs/ LLIHNs: Long-lasting insecticidal nets and hammock nets
RDT: Rapid diagnostic test
MIS: Malaria information system
PCR: Polymerase chain reaction
G6PD: Glucose-6-phosphate dehydrogenase

## References

[1] Kingdom of Cambodia. Cambodia Malaria Elimination Action Framework 2021–2025. Phnom Penh, 2020.

[2] WHO. World malaria report 2020. Geneva: World Health Organization, 2020.

[3] Kar NP, Kumar A, Singh OP, Carlton JM, Nanda N (2014). A review of malaria transmission dynamics in forest ecosystems. Parasit Vectors.

[4] Delacollette C, D'Souza C, Christophel E, Thimasarn K, Abdur R, Bell D (2009). Malaria trends and challenges in the Greater Mekong Subregion. Southeast Asian J Trop Med Public Health.

[5] Kingdom of Cambodia. Cambodia Malaria Survey 2013. Phnom Penh, 2015.

[6] Bannister-Tyrrell M, Gryseels C, Sokha S, Dara L, Sereiboth N, James N (2019). Forest goers and multidrug-resistant malaria in Cambodia: an ethnographic study. Am J Trop Med Hyg.

[7] Guyant P, Canavati SE, Chea N, Ly P, Whittaker MA, Roca-Feltrer A (2015). Malaria and the mobile and migrant population in Cambodia: a population movement framework to inform strategies for malaria control and elimination. Malar J.

[8] Canavati SE, Quintero CE, Lawford HL, Yok S, Lek D, Richards JS (2016). High mobility, low access thwarts interventions among seasonal workers in the Greater Mekong Sub-region: lessons from the malaria containment project. Malar J.

[9] Canavati SE, Chea N, Guyant P, Roca-Feltrer A, Yeung S. Strategy to address migrant and mobile populations in Cambodia. Malaria Consortium, Project Report, 2013.

[10] Kingdom of Cambodia. National Strategic Plan for Elimination of Malaria (2011–2025). Phnom Penh, 2011.

[11] Kingdom of Cambodia. Cambodia Malaria Elimination Action Framework 2016–2020. Phnom Penh, 2016.

[12] Kingdom of Cambodia. National Treatment Guidelines for Malaria in Cambodia. Phnom Penh, 2014.

[13] Malaria Consortium. Accelerating malaria elimination in northern Cambodia through early diagnosis and treatment 2020. Available from: https://www.malariaconsortium.org/media-downloads/1286/Accelerating%20malaria%20elimination%20in%20northern%20Cambodia%20through%20early%20diagnosis%20and%20treatment. Accessed 12 Oct 2020.

[14] Hustedt J, Canavati SE, Rang C, Ashton RA, Khim N, Berne L (2016). Reactive case-detection of malaria in Pailin Province, Western Cambodia: lessons from a year-long evaluation in a pre-elimination setting. Malar J.

[15] Rossi G, Van den Bergh R, Nguon C, Debackere M, Vernaeve L, Khim N (2018). Adapting reactive case detection strategies for falciparum malaria in a low-transmission area in Cambodia. Clin Infect Dis.

[16] Edwards HM, Canavati SE, Rang C, Ly P, Sovannaroth S, Canier L (2015). Novel cross-border approaches to optimise identification of asymptomatic and artemisinin-resistant *Plasmodium* infection in mobile populations crossing Cambodian borders. PLoS ONE.

[17] Lopes S, Rang C, Sarady A, Ly P, Khengthavrin B, Sovannaroth S, et al. Cross border surveillance initiatives targeting mobile and migrant populations: lessons learnt from Cambodia. Malaria Consortium, 2017.

[18] WHO. Prequalification of in vitro diagnostics programme-Product: SD BIOLINE Malaria Ag P.f/P.v. Geneva: World Health Organization, 2016.

[19] Landier J, Parker DM, Thu AM, Lwin KM, Delmas G, Nosten FH (2018). Effect of generalised access to early diagnosis and treatment and targeted mass drug administration on *Plasmodium falciparum* malaria in Eastern Myanmar: an observational study of a regional elimination programme. Lancet.

[20] Verschuere J, Decroo T, Lim D, Kindermans JM, Nguon C, Huy R (2017). Local constraints to access appropriate malaria treatment in the context of parasite resistance in Cambodia: a qualitative study. Malar J.

[21] Liverani M, Nguon C, Sok R, Kim D, Nou P, Nguon S (2017). Improving access to health care amongst vulnerable populations: a qualitative study of village malaria workers in Kampot. Cambodia BMC Health Serv Res.

[22] Lek D, Callery JJ, Nguon C, Debackere M, Sovannaroth S, Tripura R (2020). Tools to accelerate falciparum malaria elimination in Cambodia: a meeting report. Malar J.

[23] WHO. A framework for malaria elimination. Geneva: World Health Organization, 2017.

[24] Durnez L, Pareyn M, Mean V, Kim S, Khim N, Menard D (2018). Identification and characterization of areas of high and low risk for asymptomatic malaria infections at sub-village level in Ratanakiri. Cambodia Malar J.

[25] Rossi G, De Smet M, Khim N, Kindermans JM, Menard D (2017). Performance of rapid diagnostic testing in patients with suspected malaria in Cambodia, a low-endemicity country aiming for malaria elimination. Clin Infect Dis.

[26] Tripura R, Peto TJ, Veugen CC, Nguon C, Davoeung C, James N (2017). Submicroscopic *Plasmodium* prevalence in relation to malaria incidence in 20 villages in western Cambodia. Malar J.

[27] Canier L, Khim N, Kim S, Sluydts V, Heng S, Dourng D (2013). An innovative tool for moving malaria PCR detection of parasite reservoir into the field. Malar J.

[28] Kunkel A, Nguon C, Iv S, Chhim S, Peov D, Kong P (2021). Choosing interventions to eliminate forest malaria: preliminary results of two operational research studies inside Cambodian forests. Malar J.

[29] Siv S, Roca-Feltrer A, Vinjamuri SB, Bouth DM, Lek D, Rashid MA (2016). *Plasmodium vivax* malaria in Cambodia. Am J Trop Med Hyg.

[30] Roca-Feltrer A, Khim N, Kim S, Chy S, Canier L, Kerleguer A (2014). Field trial evaluation of the performances of point-of-care tests for screening G6PD deficiency in Cambodia. PLoS ONE.

